# Practical Comparison of the BioFire FilmArray Pneumonia Panel to Routine Diagnostic Methods and Potential Impact on Antimicrobial Stewardship in Adult Hospitalized Patients with Lower Respiratory Tract Infections

**DOI:** 10.1128/JCM.00135-20

**Published:** 2020-06-24

**Authors:** Blake W. Buchan, Sam Windham, Joan-Miquel Balada-Llasat, Amy Leber, Amanda Harrington, Ryan Relich, Caitlin Murphy, Jennifer Dien Bard, Samia Naccache, Shira Ronen, Amanda Hopp, Derya Mahmutoglu, Matthew L. Faron, Nathan A. Ledeboer, Amanda Carroll, Hannah Stone, Oluseun Akerele, Kathy Everhart, Andrew Bonwit, Christina Kwong, Rebecca Buckner, Del Warren, Randal Fowler, Sukantha Chandrasekaran, Holly Huse, Shelley Campeau, Romney Humphries, Corrin Graue, Angela Huang

**Affiliations:** a The Medical College of Wisconsin, Milwaukee, Wisconsin, USA; b The Ohio State University, Columbus, Ohio, USA; c Nationwide Children’s Hospital, Columbus, Ohio, USA; d Loyola University Medical Center, Maywood, Illinois, USA; e Indiana University School of Medicine, Indianapolis, Indiana, USA; f University of Nebraska Medical Center, Omaha, Nebraska, USA; g Children’s Hospital of Los Angeles, Los Angeles, California, USA; h University of California Los Angeles, Los Angeles, California, USA; i BioFire Diagnostics, LLC, Salt Lake City, Utah, USA; j Froedtert Hospital, Milwaukee, Wisconsin, USA; UNC School of Medicine

**Keywords:** multiplex, stewardship, medical outcomes, pneumonia

## Abstract

Lower respiratory tract infections, including hospital-acquired and ventilator-associated pneumonia, are common in hospitalized patient populations. Standard methods frequently fail to identify the infectious etiology due to the polymicrobial nature of respiratory specimens and the necessity of ordering specific tests to identify viral agents. The potential severity of these infections combined with a failure to clearly identify the causative pathogen results in administration of empirical antibiotic agents based on clinical presentation and other risk factors. We examined the impact of the multiplexed, semiquantitative BioFire FilmArray Pneumonia panel (PN panel) test on laboratory reporting for 259 adult inpatients submitting bronchoalveolar lavage (BAL) specimens for laboratory analysis. The PN panel demonstrated a combined 96.2% positive percent agreement (PPA) and 98.1% negative percent agreement (NPA) for the qualitative identification of 15 bacterial targets compared to routine bacterial culture. Semiquantitative values reported by the PN panel were frequently higher than values reported by culture, resulting in semiquantitative agreement (within the same log_10_ value) of 43.6% between the PN panel and culture; however, all bacterial targets reported as >10^5^ CFU/ml in culture were reported as ≥10^5^ genomic copies/ml by the PN panel. Viral targets were identified by the PN panel in 17.7% of specimens tested, of which 39.1% were detected in conjunction with a bacterial target. A review of patient medical records, including clinically prescribed antibiotics, revealed the potential for antibiotic adjustment in 70.7% of patients based on the PN panel result, including discontinuation or de-escalation in 48.2% of patients, resulting in an average savings of 6.2 antibiotic days/patient.

## Abstract (precis)

Lower respiratory tract infections, including hospital-acquired and ventilator-associated pneumonia, are common in hospitalized patient populations. Standard methods frequently fail to identify the infectious etiology due to the polymicrobial nature of respiratory specimens and the necessity of ordering specific tests to identify viral agents. The potential severity of these infections combined with a failure to clearly identify the causative pathogen results in administration of empirical antibiotic agents based on clinical presentation and other risk factors.

## INTRODUCTION

Lower respiratory tract infections, including community-acquired pneumonia (CAP), hospital-acquired pneumonia (HAP), and ventilator-associated pneumonia (VAP), are linked to significant morbidity and mortality (1–4). These infections can be caused by bacterial, viral, or fungal agents, depending on patient exposure and clinical risk factors. Bacterial pathogens associated with HAP and VAP, including Staphylococcus aureus, Pseudomonas aeruginosa, Klebsiella pneumoniae, and other members of the *Enterobacteriaceae*, are often multidrug resistant (2). This includes carbapenemase-producing organisms, which have been independently associated with higher mortality rates following infection (5). Selected studies have identified a significant decrease in mortality for patients who receive prompt and effective antibiotic therapy to treat pneumonia (3, 4, 6). Based on these findings, broad-spectrum empirical antibiotic therapy consisting of 2 or 3 agents is recommended for patients with symptoms consistent with HAP or VAP until definitive laboratory results are available to inform targeted and specific therapy (3, 7). As a result, it is estimated that up to 50% of antibiotics used in hospital intensive care units (ICUs) are prescribed to treat these infections (3, 8). An emphasis of the current American Thoracic Society (ATS) and Infectious Disease Society of America (IDSA) guidelines for management of patients with HAP or VAP is to reduce exposure to broad-spectrum and unnecessary antibiotics by targeting therapy to treat the most likely pathogens based on patient risk factors and laboratory results (3).

Laboratory identification of a specific infectious etiology in patients with pneumonia has been associated with a statistically significant reduction in mortality, presumably because it enables targeted effective therapy (6). Historically, quantitative and qualitative bacterial culture has been the primary approach for laboratory diagnosis of lower respiratory tract infections, including pneumonia. These methods are useful in establishing definitive antibiotic therapy; however, recovery of potential pathogens is variable due to antibiotic exposure prior to specimen collection, fastidious growth characteristics of some pathogens, or overgrowth of resident flora (9). These factors contribute to variable culture sensitivity and turnaround times of 48 h or more. Furthermore, additional specific culture or molecular tests are required to identify atypical bacteria or viral pathogens, and these tests may not be routinely ordered by clinicians. Combined, these shortcomings diminish the ability of current standard-of-care (SOC) methods to rapidly and accurately identify specific infectious etiologies and target antibiotic therapy for these infections.

Molecular diagnostics, including PCR-based tests, generate a sensitive result within hours of specimen collection. These tests have the potential to reduce the duration of broad-spectrum empirical antibiotic therapy by identifying pathogenic organisms or specific antibiotic resistance markers 2 to 3 days sooner than routine methods. Specifically, rapid molecular detection of methicillin-resistant S. aureus (MRSA) in respiratory specimens has been associated with a potential 60% to 80% reduction in anti-MRSA antibiotic days and a total savings of >$60,000 in antibiotic cost annually (10). Multiplexed molecular tests, including the ePlex RP (GenMark Diagnostics) and the BioFire FilmArray Respiratory 2 (RP2) panel (BioFire Diagnostics), enable broad detection of viral pathogens in respiratory specimens but identify only a few bacteria, including Mycoplasma pneumoniae, Chlamydia pneumoniae, and *Bordetella* species (11).

The FilmArray Pneumonia panel (PN panel; BioFire Diagnostics, LLC) is a sample-to-answer PCR-based *in vitro* diagnostic test that analyzes native (untreated) sputum (including endotracheal aspirates) and bronchoalveolar lavage (BAL) (including mini-BAL) specimens for the presence of bacteria, viruses, and genetic markers of antimicrobial resistance within approximately 75 min, with <5 min of hands-on time. The PN panel reports qualitative (“detected” or “not detected”) results for viral targets, bacterial targets associated with atypical pneumonia, and antibiotic resistance markers while providing a semiquantitative value for 15 additional bacterial targets commonly associated with respiratory infections (Table 1). Semiquantitative reporting is intended to facilitate interpretation of results based on the absolute and relative abundance of each target detected. This is consistent with current culture-based laboratory protocols for analysis and reporting of respiratory specimens proposed by the IDSA and others to aid in discrimination of true infection from clinically insignificant colonization of the airway (3, 12, 13). These culture-based guidelines propose specimen-specific thresholds ranging from 10^4^ CFU/ml for BAL specimens to 10^5^ to 10^6^ CFU/ml for endotracheal aspirates (ETA) and sputa to define clinically significant infection and minimize reporting of low-abundance, likely commensal organisms to reduce the use of potentially unnecessary antibiotics. Similarly, the PN panel also has a detection threshold intended to prevent reporting of bacteria present at low levels in respiratory specimens.

**TABLE 1:** BioFire PN panel targets

Category (result type)	Target
Viruses (qualitative)	Adenovirus
	Coronavirus
	Human metapneumovirus
	Human rhinovirus/enterovirus
	Influenza A virus
	Influenza B virus
	Parainfluenza virus
	Respiratory syncytial virus
	Bacteria (qualitative result)
Bacteria (qualitative)	Chlamydia pneumoniae
	Legionella pneumophila
	Mycoplasma pneumoniae
Bacteria (semiquantitative^*a*^)	Acinetobacter calcoaceticus-A. baumannii complex
	Enterobacter cloacae complex
	Escherichia coli
	Haemophilus influenzae
	Klebsiella aerogenes
	Klebsiella oxytoca
	Klebsiella pneumoniae group
	Moraxella catarrhalis
	*Proteus* spp.
	Pseudomonas aeruginosa
	Serratia marcescens
	Staphylococcus aureus
	Streptococcus agalactiae
	Streptococcus pneumoniae
	Streptococcus pyogenes
Antimicrobial resistance markers (qualitative, conditionally reported)	
Carbapenemases	KPC^*b*^
	NDM^*b*^
	IMP^*b*^
	VIM^*b*^
	OXA-48-like^*c*^
Extended-spectrum beta-lactamases	CTX-M^*b*^
Methicillin resistance genes	*mecA*/*mecC* and MREJ^*d*^

a Reported as 10^4^, 10^5^, 10^6^, or >10^7^ copies/ml.

b Reported when A. calcoaceticus-A. baumannii complex, E. cloacae complex, E. coli, K. aerogenes, K. oxytoca, K. pneumoniae group, *Proteus* spp., P. aeruginosa, or S. marcescens is also detected.

c Reported when E. cloacae complex, E. coli, K. aerogenes, K. oxytoca, K. pneumoniae group, *Proteus* spp., or S. marcescens is also detected.

d Reported when S. aureus is also detected.

The analytical and clinical performance (accuracy) of the PN panel was established based on data collected from over 1,600 clinical BAL and sputum specimens collected across 8 U.S. medical centers during the clinical evaluation that was performed for regulatory clearance (14). The aim of this study was to conduct a practical analysis of a subset of those specimens comparing results reported using routine SOC methods to those obtained using the PN panel and to assess the potential impact of the PN panel results on antibiotic utilization in these patients. Specifically, we compared positivity rates in terms of total positive specimens and total targets detected using SOC versus the PN panel, as well as the absolute and relative concordance of quantitative results for identified bacterial targets. We also noted the incidence of viral targets detected by the PN panel compared to clinician-ordered SOC viral tests. Finally, we conducted a retrospective chart review to assess the potential impact of the PN panel results on antibiotic modifications and stewardship.

## MATERIALS AND METHODS

### Enrollment of specimens.

Clinical specimens (bronchoalveolar lavage [BAL], mini-BAL, and endotracheal aspirate [ETA] specimens and sputum from subjects of all ages and multiple care settings) were enrolled at eight U.S. clinical centers between October 2016 and July 2017 as part of the clinical trial for regulatory clearance of the PN panel (14).

For this study, subsets of the clinical trial specimens comprising 259 residual BAL (*n* = 237) or mini-BAL (*n* = 22) specimens were selected for further analysis. BAL is a bronchoscopy-guided procedure which installs 100 to 250 ml of saline for specimen collection, whereas mini-BAL uses a non-bronchoscopy-guided “blind” lavage catheter and installs approximately 20 ml of saline into the lung. Specimens were specifically selected from adult inpatients, since this population is at highest risk for HAP and VAP and frequently receives broad-spectrum empirical antibiotic therapy for presumptive respiratory tract infections, including those caused by multidrug-resistant pathogens. Sputum and endotracheal aspirates were specifically excluded from the analysis. Based on the full clinical trial set, sputum and endotracheal aspirate specimens were over three times as likely as BAL or mini-BAL specimens to have ≥3 targets detected (51 BAL and 162 sputum specimens) and almost twice as likely to have bacterial targets that were not recovered by specialized reference culture methods (328 BAL and 547 sputum specimens) (14). As a result of this added complexity, we chose to focus on the analysis of BAL and mini-BAL specimens in the current study.

The final cohort included randomly selected specimens from each study site proportional to the total specimens enrolled at that site. This included specimens submitted to the Medical College of Wisconsin, Milwaukee, WI (*n* = 49); The Ohio State University Wexner Medical Center, Columbus, OH (*n* = 52); Nationwide Children’s Hospital, Columbus, OH (*n* = 1); Loyola University Medical Center, Maywood, IL (*n* = 47); Indiana University School of Medicine, Indianapolis, IN (*n* = 39); University of Nebraska Medical Center, Omaha, NE (*n* = 28); and University of California Los Angeles Health, Los Angeles, CA (*n* = 43). The PN panel result was compared to the SOC result(s) reported by each clinical laboratory to assess the impact of the PN panel on result reporting, positivity, and the potential for antibiotic stewardship. The average age of enrolled patients was 57.7 years (range, 18 to 92 years), and 50.6% were male. All SOC and PN panel testing was initiated within 24 h of collection at the study enrollment site. Each clinical site followed its own routine procedures for defining specimen acceptability criteria, selection of culture medium, culture workup, bacterial identification, susceptibility testing, and result reporting (site-specific methods are detailed below).

### Chart review and data abstraction.

Clinical, demographic, and laboratory data were abstracted from the laboratory information system (LIS) and electronic health record (EHR) at each clinical site by individuals who were not involved in either clinical testing or PN panel testing of specimens and who were blinded to PN panel results. The LIS was reviewed to identify clinician-ordered tests and SOC results associated with each submitted specimen, including bacterial culture, antimicrobial susceptibility tests (ASTs), and molecular tests for viral agents that were ordered as part of clinical care. SOC testing results encompassed all laboratory results reported from the same specimen tested with the PN panel, as well as other respiratory specimens collected within ±24 h of the enrolled specimen to provide a comprehensive comparison. The EHR was reviewed to retrieve clinical and demographic data, including hospitalization status at the time of specimen collection, subject age, and subject gender, and to ascertain antibiotic use from 7 days preceding to 14 days following specimen collection. Abstracted LIS and EHR data were used to assess antibiotic adjustments based on SOC results and to determine the potential impact of the PN panel results according to criteria described below.

A waiver of the requirement for informed consent was obtained from the institutional review board (IRB) at each study site for the use of residual specimens and for the abstraction and deidentification of subject information from the laboratory and medical record.

### BioFire PN panel.

Testing of specimens using the PN panel was conducted at each site in accordance with the manufacturer’s instructions for use and site-specific laboratory protocols to ensure safe handling of specimens and maintenance of quality. All primary specimens were handled within in a biological safety cabinet (BSC) during processing for PN panel testing. A flocked swab (provided) was used to sample each BAL specimen and inoculate a FilmArray injection vial (FAIV) containing sample buffer. Inoculated FAIVs were capped and inverted three times to facilitate organism release, and contents were then injected into the PN panel test pouch. Inoculated pouches were inserted into the FilmArray instrument for analysis (∼75-min run time). Each specimen was processed separately, and the BSC was surface disinfected prior to processing subsequent specimens.

The PN panel test pouch contains all reagents necessary for specimen lysis, nucleic acid extraction, reverse transcription, amplification, and detection of genomic sequences unique to each of the 33 panel targets (Table 1). In addition, the test pouch contains two internal controls to assess pouch function and enable the calculation of semiquantitative results. If either internal control fails, a result of “invalid” is reported. For viral and atypical bacterial targets, a qualitative result of either “detected” or “not detected” is reported. For bacterial targets reported semiquantitatively, the PN panel reports the specific target with a log_10_ binned value of 10^4^, 10^5^, 10^6^, or >10^7^ genomic copies/ml; targets quantified by the internal software at 10^3.5^ to 10^4.5^ copies/ml are reported as 10^4^ copies/ml; targets quantified at 10^4.5^ to 10^5.5^ copies/ml are reported as 10^5^ copies/ml, etc. Targets quantified at <10^3.5^ copies/ml are reported as “not detected.” Genetic markers of antimicrobial resistance are reported qualitatively as “detected” or “not detected” only if a commensurate bacterial target is detected and reported (Table 1).

### Standard-of-care testing.

Standard-of-care testing at each of the seven clinical centers was conducted in accordance with clinician test orders and local laboratory protocols. Bacterial culture was conducted by inoculating a portion of the BAL fluid into several selective and differential growth media, including blood agar, chocolate agar, MacConkey agar, Columbia colistin-nalidixic acid (CNA) agar, and *Haemophilus* isolation agar, in accordance with site-specific protocols. Plates were inoculated and streaked using calibrated loops in accordance with local protocols to achieve a semiquantitative culture result. Inoculated media were incubated at 35°C in a 5% CO_2_ atmosphere and were examined daily for bacterial growth. Bacterial colonies were identified using matrix-assisted laser desorption ionization-time of flight mass spectrometry (MALDI-TOF MS) as the primary method, supplemented with a combination of manual biochemical tests and automated phenotypic identification systems when necessary in accordance with local protocols. Potential pathogens and normal flora were reported semiquantitatively in CFU per milliliter by 6 of 7 laboratories; a single laboratory reported semiquantitative results using descriptors (e.g., “rare,” “few,” “moderate,” or “many”). Potential pathogens quantified at <10^4^ CFU/ml (or “rare”) were not routinely reported unless they were in pure culture or were associated with polymorphonuclear leukocytes (PMNs) in the primary Gram stain. Antibiotic susceptibility tests (ASTs) were conducted using automated AST instruments, including the Vitek-2 (bioMérieux, Durham, NC) and BD Phoenix (BD, Sparks, MD). Additional phenotypic tests, including disk diffusion or Etest (bioMérieux, Durham, NC), were used when necessary (e.g., when automated AST failed or to determine the presence of extended-spectrum beta-lactamase) in accordance with local protocols. Molecular tests for viral pathogens were conducted using FDA-cleared (GenMark eSensor XT-8, Luminex Verigene RPP, or the BioFire FilmArray Respiratory [RP] panel) and laboratory-developed assays at each site upon clinical order.

### Assessment of the potential impact of PN panel results on antibiotic therapy and stewardship.

The potential impact of PN panel results on antibiotic therapy and stewardship was assessed based on a comparison of the PN panel results to the results of the clinician-ordered SOC tests performed on each BAL or mini-BAL specimen. It was assumed that all patients with a clinical order for microbiologic testing on a BAL or mini-BAL specimen were being evaluated for diagnosis of pneumonia and that antimicrobials were not being used to treat any concomitant infection unless otherwise indicated by chart review. Antimicrobials targeting anaerobic pathogens (e.g., metronidazole) and those not used for treatment of pneumonia (e.g., daptomycin) were not included in the analysis. SOC BAL specimen culture results were considered the gold standard and were used to define potential modifications based on the PN panel result as inappropriate or appropriate. If a diagnostic test was not performed as part of SOC, but a potential pathogen was detected by the PN panel (e.g., the PN panel was positive for influenza virus but no SOC PCR for influenza virus was performed), the PN panel result was considered correct. This rationale was used based on the observed >93% sensitivity and >99% specificity of the PN panel for identification of viral targets in BAL specimens compared to conventional PCR tests (followed by sequencing) for each analyte (14).

Potential therapy modifications were classified as (i) appropriate antibiotic escalation, (ii) appropriate antibiotic de-escalation, (iii) inappropriate antibiotic escalation or continuation, (iv) inappropriate antibiotic de-escalation, or (v) no change, in accordance with definitions and examples provided in Table S1. Antibiotic escalations were defined as any broadening of antimicrobial spectrum, which could include a change in agent (e.g., ceftriaxone to cefepime) or the addition of an agent(s). Antibiotic de-escalation was defined as any narrowing of antimicrobial spectrum, which could include a change in agent (e.g., cefepime to ceftriaxone) or discontinuation of an agent(s) in a multidrug regimen. It was possible for a single patient to qualify for more than one potential intervention, depending on the number of organisms identified, specific organism identification, and empirical antibiotic therapy regimen.

The time of specimen collection, time of final culture result, and time(s) of antibiotic initiation or discontinuation were collected and used to categorize the potential antibiotic modifications. The time of the PN panel result was set as 4 h from the time of specimen collection, based on a combined estimate of specimen transport time, in-lab processing, and PN panel run time (approximately 75 min). If empirical antibiotics were initiated, it was assumed that these could be discontinued based on concordant negative PN panel and culture results as soon as the PN panel result was available. If both PN panel and SOC results yielded the same clinically relevant organism(s), it was assumed that antibiotics could be initiated, escalated, or de-escalated to optimal therapy as soon as PN panel results became available. The number of hours of antibiotic saved or hours earlier that antibiotics could have been initiated or escalated was calculated based on the difference between the time of PN panel result (i.e., 4 h after specimen collection) and the actual time of antibiotic change, which was obtained from chart review and was assumed to be based on the SOC culture and/or PCR result(s). If discontinuation times for antibiotics were not found in chart review, they were still included in the analysis of potential escalation or de-escalation but were excluded when the number of antibiotic hours saved or number of hours sooner that appropriate antibiotics could have been initiated was calculated.

### Statistical analysis.

Clinical performance of the PN panel, including sensitivity and specificity, was calculated using standard methods, including binomial two-sided 95% confidence intervals (95% CI), which were calculated in accordance with methods described by Newcombe (15).

## RESULTS

### Impact of the PN panel on overall specimen and target positivity.

**FIG 1:**
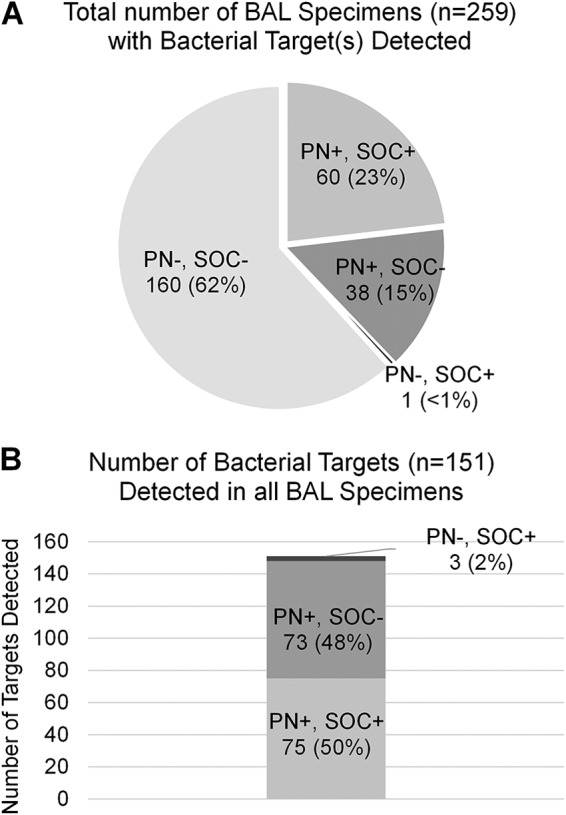
The BioFire PN panel (PN) identified at least one bacterial target in 63% more BAL specimens than standard-of-care culture (SOC) (A) and identified nearly twice as many total bacterial targets as SOC culture (B).

Bacterial culture was ordered for all 259 BAL specimens evaluated in this study. Among these, 60 (23.2%) specimens were reported as positive for at least one on-panel bacterial target by both culture and the PN panel. An additional 38 (14.7%) specimens were reported as positive for at least one bacterial target by the PN panel but had culture results reported as “negative/no growth” (15/38; 39.5%) or “negative/normal oral flora” (23/38; 60.5%) by the reporting laboratory (Fig. 1A). Across all specimens tested, a total of 75 individual bacterial targets were detected by both the PN panel and culture. An additional 73 targets were detected by the PN panel alone, and 3 on-panel targets were detected by culture alone (Fig. 1B). These data demonstrate a 63.3% increase in the number of BAL specimens reported as positive and a 94.8% increase in individual on-panel targets reported when the PN panel was compared to routine bacterial culture. Off-panel bacterial targets were reported by culture in 30 (11.6%) specimens. These included Haemophilus parainfluenzae (*n* = 1), Burkholderia cepacia (*n* = 1), Morganella morganii (*n* = 1), Providencia stuartii (*n* = 1), *Lactobacillus* sp. (*n* = 1), *Corynebacterium* spp. (*n* = 2), *Achromobacter* spp. (*n* = 2), viridans group streptococci (*n* = 3), *Enterococcus* spp. (*n* = 3), “beta-hemolytic *Streptococcus* not type A” (*n* = 3), coagulase-negative staphylococci (*n* = 3), and other bacteria that were not definitively identified by the reporting clinical laboratory (*n* = 9). Many of these targets are not considered respiratory pathogens unless they are in pure cultures or predominant; however, 17/30 (56.6%) were quantified at ≥10^4^ CFU/ml and may have been clinically significant.

**FIG 2:**
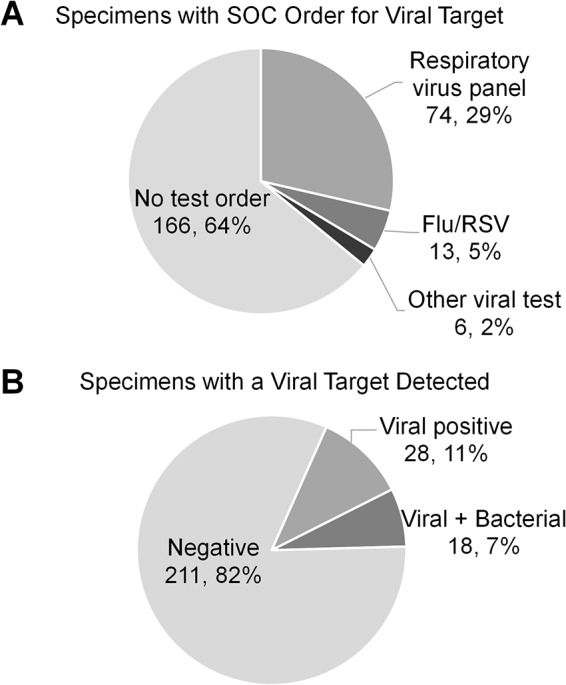
Molecular tests for viral pathogens were clinically ordered for only 93/259 (35.9%) BAL specimens submitted for bacterial culture and included primarily multiplexed respiratory panel tests (A). At least one viral target was detected by the PN panel in 46/259 (17.7%) BAL specimens, either alone or in addition to bacterial targets (B). Only 11/46 (23.9%) specimens with a positive viral detection by the PN panel had a clinician-ordered molecular test for viral pathogens.

Molecular tests for viral pathogens were clinically ordered for only 93/259 (35.9%) BAL specimens submitted for bacterial culture and included primarily multiplexed respiratory panel tests (*n* = 74) and influenza virus or respiratory syncytial virus (RSV) tests (*n* = 13) (Fig. 2A). The SOC positivity rate was 14/93 (15.1%) among specimens with a clinical order for viral-pathogen testing. At least one viral target was detected by the PN panel in 46/259 (17.7%) BAL specimens. Among these, 18/46 (39.1%) were also positive for at least one bacterial target by the PN panel (Fig. 2B). Only 11/46 (23.9%) specimens with a positive viral detection by the PN panel had a clinician-ordered molecular test for viral pathogens, demonstrating that a large proportion of viral targets go undetected due to a lack of corresponding test orders.

### Qualitative agreement of the PN panel with routine culture for bacterial targets.

An assessment of the overall qualitative performance of the PN panel for detection of bacterial targets demonstrated 96.2% (75/78) positive percent agreement (PPA) and 98.1% negative percent agreement (NPA) (3,734/3,807) with routine culture results (Table 2). Notably, these data are similar to the sensitivity reported for BAL specimens compared to an expanded quantitative reference culture method (98.1%) and reported SOC results (92.3%) in the clinical trial study (14). All 3 culture-positive, PN panel-negative targets were detected at a low quantity in the BAL specimens. They included (i) E. coli reported at 10^3^ CFU/ml in a culture also containing S. aureus at 10^4^ CFU/ml and K. pneumoniae at 10^3^ CFU/ml (both S. aureus and K. pneumoniae were detected by the PN panel), (ii) S. aureus reported at 10^3^ CFU/ml in culture (the PN panel detected Streptococcus agalactiae), and (iii) P. aeruginosa reported as “few” in culture (the PN panel was negative for all targets). It is possible that each of these organisms was present at a concentration below the PN panel threshold for reporting a positive result, which was specifically designed to be 10^3.5^ genomic copies/ml in accordance with current guidelines to reduce the risk of reporting clinically insignificant results in BAL specimens (3, 12). Laboratories adhering to these guidelines may not routinely report the presence organisms at this quantity in cultured BAL specimens.

**TABLE 2:** Qualitative detection of bacterial targets between the BioFire PN panel and standard-of-care culture^*a*^

Target	No. of samples				PPA (CI)	NPA (CI)
	SOC+ PN+	SOC+ PN−	SOC− PN+	SOC− PN−		
A. baumannii complex	1	0	0	258	100 (6–100)	100 (98–100)
E. cloacae complex	7	0	4	248	100 (56–100)	98.4 (96–99)
K. aerogenes	3	0	1	255	100 (31–100)	99.6 (98–100)
E. coli	1	1^*b*^	1	254	50.0 (3–97)	99.6 (97–100)
H. influenzae	4	0	16	239	100 (40–100)	93.7 (90–96)
K. oxytoca	2	0	3	254	100 (20–100)	98.8 (96–99)
K. pneumoniae group	8	0	3	248	100 (60–100)	98.8 (96–100)
M. catarrhalis	2	0	6	249	100 (20–100)	96.9 (95–99)
*Proteus* spp.	2	0	2	254	100 (20–100)	99.2 (97–100)
P. aeruginosa	18	1^*c*^	6	234	94.7 (72–100)	97.5 (94–99)
S. marcescens	3	0	0	256	100 (31–100)	100 (96–100)
S. agalactiae	1	0	6	253	100 (5–100)	97.6 (94–99)
S. pneumoniae	2	0	3	254	100 (20–100)	98.8 (96–100)
S. pyogenes	0	0	1	258	ND	99.6 (98–100)
S. aureus	21	1^*d*^	21	216	95.5 (75–100)	91.1 (87–94)
Total	75	3	73	3,885	96.2 (88–99)	98.1 (98–99)

a SOC, standard-of-care culture; PN, BioFire PN panel; CI, 95% confidence interval; PPA, positive percent agreement; NPA, negative percent agreement.

b E. coli reported as 1 × 10^3^ in routine culture in addition to 1 × 10^3^ K. pneumoniae and 5 × 10^4^ S. aureus, both of which were detected by the PN panel.

c P. aeruginosa reported as “few” in routine culture.

d S. aureus reported at 4 × 10^3^ CFU/ml in routine culture.

**FIG 3:**
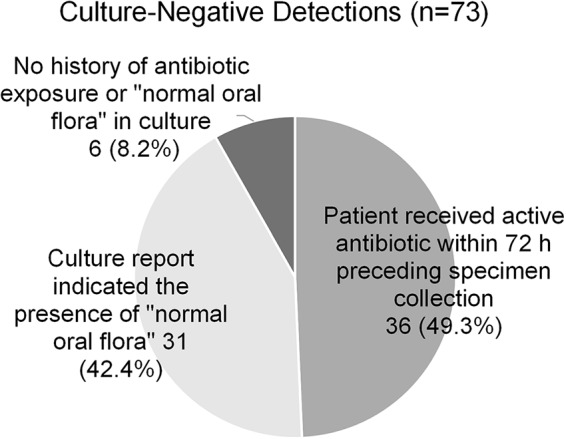
In approximately half of all culture-negative PN panel detections, the patient had received antibiotic therapy in the 72 h preceding specimen collection, which could contribute to failure to recover these bacteria in culture. An additional 43% of specimens with culture-negative deductions reported the presence of normal oral flora in the culture.

The PN panel identified 73 bacterial targets that were not reported by routine culture. The most common culture-negative detections were S. aureus (*n* = 21) and H. influenzae (*n* = 16), though at least one culture-negative detection was observed for all but two of the PN panel bacterial targets (Table 2). A chart review revealed that 36 (49.3%) of these culture-negative detections were made in specimens obtained from patients that had received at least one dose of an antibiotic with potential activity against the specific bacterial target within the 72 h preceding specimen collection (Fig. 3). An additional 31 (42.4%) culture-negative detections had a routine culture report indicating the presence of “normal oral flora,” which could have obscured detection and reporting of these bacterial targets in culture. Furthermore, 10 (32.3%) of these detections were quantified at 10^4^ genomic copies/ml by the PN panel, indicating a low concentration of target that may have been overlooked or disregarded during routine culture workup of specimens containing significant normal oral flora. The remaining 6 (8.2%) culture-negative detections were in samples from patients without antibiotic exposure and in cultures without a report of “normal oral flora.” In 3/6 (50%), the target was quantified as 10^4^ genomic copies/ml by the PN panel and may have been below the culture threshold for detection or reporting. The final 3 culture-negative detections were reported at 10^5^ or 10^6^ genomic copies/ml by the PN panel.

### Semiquantitative agreement of the PN panel bacterial targets with routine culture.

Semiquantitative results of the PN panel (number of genomic copies per milliliter) and culture (CFU per milliliter) were compared (Table 3). Among positive cultures, concordance between the PN panel and culture quantitation was 43.6% (34/78). Concordance was poorest when bacterial culture enumeration was low; only 18.8% (3/16) concordance was observed for bacteria quantified in culture at 10^3^ CFU/ml (concordance defined as “not detected” by the PN panel) and 11.4% (4/35) concordance for bacteria quantified at 10^4^ CFU/ml in culture (concordance defined as 10^4^ copies/ml reported by the PN panel). In all cases of discordant quantitation, the PN panel result was higher than that of culture, with 77.2% (34/44) of these results exceeding culture quantitation by >1 log. Notably, all 27 bacterial targets reported as >10^5^ CFU/ml in culture were reported as 10^5^, 10^6^, or ≥10^7^ genomic copies/ml by the PN panel (100% concordance), with 85.2% (23/27) reported as ≥10^7^ genomic copies/ml. These data demonstrate a strong bias toward higher semiquantitative values when the PN panel was compared to routine culture results for on-panel targets.

**TABLE 3:** Quantitative agreement of bacterial targets

PN panel result (copies/ml)	No. of samples^*a*^ with SOC culture result (CFU/ml)			
	Not detected	10^3^	10^4^	≥10^5^
Not detected	3,734	3	0	0
10^4^	24 (8)	6	4	0
10^5^	27 (17)	3	4	1
10^6^	9 (4)	2	12	3
≥10^7^	13 (7)	2	15	23
% concordant^*b*^	98.1 (3,734/3,807)	18.8 (3/16)	11.4 (4/35)	100 (27/27)

a Numbers in parentheses are the numbers of culture-negative results obtained for specimens from patients who received antibiotics with potential activity against the given bacterial target detected within 72 h preceding specimen collection. One laboratory reported bacterial culture quantitation (11 isolates) as “few,” “moderate,” or “many”; these were categorized as 10^3^, 10^4^, and ≥10^5^ CFU/ml, respectively. Shading indicates concordance between the BioFire PN panel and routine culture quantitation.

b Concordance between the PN panel and culture quantitation among all positive cultures was 43.6% (34/78).

The PN panel and culture were 98.1% (3,734/3,807) concordant for individual bacterial targets among cultures reported as negative for on-panel targets (concordance defined as culture negative and “not detected” by the PN panel). The 73 culture-negative targets detected by the PN panel were reported at concentrations ranging from 10^4^ to ≥10^7^ genomic copies/ml. Nearly 50% (36/73) of these detections were in specimens obtained from patients who had received potentially effective antibiotic therapy within 72 h preceding specimen collection.

### Correlation of the PN panel and culture in identifying the predominant bacterial target in BAL specimens.

In addition to semiquantitative concordance, we investigated the concordance of results based on relative abundance of targets detected by the PN panel and culture (i.e., rank order of targets detected in a specimen). At least one bacterial target was detected by both the PN panel and routine culture in 59 specimens, including 30 specimens with multiple bacterial targets (2 to 6) reported by the PN panel and/or culture.

The overall concordance between the PN panel and culture for reporting the same bacterial target as most predominant in a given specimen was 93.2% (55/59) (Table 4). This included 100% concordance for all 29 specimens with a single target detected by both the PN panel and culture and 86.7% (26/30) for specimens with multiple targets detected by the PN panel and/or culture. Two of the four discordant results occurred in specimens with one target reported by culture and two reported by the PN panel. In both cases, the PN panel reported S. aureus as the predominant target at a concentration 1 log_10_ higher than Enterobacter cloacae, whereas culture reported only the E. cloacae (i.e., no S. aureus present). A chart review revealed that both patients had received antistaphylococcal antibiotics within 72 h preceding specimen collection, which may have accounted for the culture-negative result. A third discordant result occurred in a specimen that was reported as positive for Streptococcus pneumoniae and P. aeruginosa by both the PN panel and culture; however, the PN panel reported S. pneumoniae as predominant (S. pneumoniae, 10^7^ copies/ml; P. aeruginosa, 10^5^ copies/ml), while culture reported P. aeruginosa as predominant (P. aeruginosa, 10^4^ CFU/ml; S. pneumoniae, “few”). This patient had no record of receiving antibiotics prior to specimen collection. The discordance in predominant target may be due to a difference in growth rate, poor recovery of these bacteria in culture, or inaccurate molecular quantitation by the PN panel. Notably, analytic studies assessing the accuracy of the PN panel quantitation using quantified reference standards demonstrated 97.2% accuracy within the defined log_10_ bins, suggesting that this discordance was likely related to the fastidious growth characteristics of S. pneumoniae and failure to accurately quantify in culture (14). The final discordant result occurred in a specimen that was reported as positive for Klebsiella oxytoca, Serratia marcescens, P. aeruginosa, and S. aureus by both the PN panel and culture. K. oxytoca, S. marcescens, and P. aeruginosa were all quantified at equivalent 10^4^ copies or CFU/ml by the PN panel and culture, respectively; however, S. aureus was reported as predominant by the PN panel (10^5^ copies/ml), whereas culture reported S. aureus as the least abundant target in the specimen (10^3^ CFU/ml). This patient had no record of receiving antibiotics prior to specimen collection.

**TABLE 4:** Correlation of predominant bacterial target detected in 59 culture-positive BAL specimens

No. of targets detected with the PN panel	Detection rate^*a*^ for number of targets detected by culture (%)			
	1	2	3	4
1	29/29 (100)			
2	11/13^*b*^ (84.6)	8/9^*c*^ (88.9)	1/1 (100)	
3	1/1 (100)	2/2 (100)		
4	1/1 (100)	1/1 (100)		0/1^*d*^ (0)
5				
6	1/1 (100)			

a Values are given as (number of BAL specimens with the same bacterial target reported as predominant by PN panel and culture)/(number of BAL specimens with the indicated number of bacterial and PN panel detections).

b One specimen was reported as 10^6^ S. aureus and 10^5^ E. cloacae by the PN panel with only 10^5^ E. cloacae reported in culture, and one specimen was reported as 10^5^ S. aureus and 10^4^ E. cloacae by the PN panel with only 10^4^ E. cloacae reported in culture.

c One specimen was reported as 10^7^ S. pneumoniae and 10^5^ P. aeruginosa by the PN panel while culture reported 10^4^ P. aeruginosa and “few” S. pneumoniae.

d One specimen was reported as 10^5^ S. aureus and 10^4^ K. oxytoca, S. marcescens, and P. aeruginosa by the PN panel while culture reported 10^4^ K. oxytoca, S. marcescens, and P. aeruginosa and only 10^3^ S. aureus.

### Detection of markers of antibiotic resistance.

The presence of genetic resistance markers is reported conditionally by the PN panel, only for specimens with a commensurate bacterial pathogen detected (Table 1). S. aureus was detected in 42 specimens by PN panel, of which 21 (50%) were positive for S. aureus in corresponding cultures. Among 18 isolates with routine susceptibility results available, the PN panel demonstrated 81.8% (9/11) sensitivity and 85.7% (6/7) specificity for the identification of MRSA (based on detection both of *mecA* and *mecC* and of the staphylococcal cassette chromosome *mec* [SCC*mec*] right-extremity junction [MREJ]). The two specimens yielding false-negative results contained MRSA based on phenotypic susceptibility results at 5.0 × 10^3^ and 1.5 × 10^4^ CFU/ml in culture, while the PN panel reported S. aureus at 10^5^ copies/ml, with *mecA*/*mecC* and MREJ reported as “not detected” (i.e., methicillin-susceptible S. aureus [MSSA]), for both specimens. These performance characteristics are somewhat lower than what was observed in the clinical trial data set, which reported postresolution sensitivity of 88.9% and specificity of 91.4% based on combined phenotypic and molecular detection of MRSA in BAL specimens (14). This is similar to the reported sensitivity (91% to 94%) and specificity (71% to 98%) of molecular tests designed to specifically detect MRSA in nasal specimens, as well as results obtained using these tests to detect MRSA in BAL specimens (16–18).

The PN panel reported the presence of a carbapenemase gene (*bla*_KPC_, *bla*_NDM_, *bla*_VIM_, *bla*_IMP_, or *bla*_OXA-48_) or an extended-spectrum beta-lactamase (ESBL) gene (*bla*_CTX-M_) in 4 of 53 specimens containing a commensurate bacterial target. This included two specimens reported positive for *bla*_KPC_ and one specimen each for *bla*_NDM_ and *bla*_CTX-M_ (Table 5). A phenotypically carbapenem-resistant organism was reported in routine culture for both specimens that tested positive for *bla*_KPC_. Specimen B-01-015 was positive for *bla*_NDM_ by the PN panel but yielded only carbapenem-susceptible E. cloacae in culture. Specimen B-08-056 was positive for *bla*_CTX-M_ by the PN panel and yielded a carbapenem-resistant P. aeruginosa isolate in culture. No specific resistance marker was identified by the PN panel in three specimens containing carbapenem-resistant P. aeruginosa (B-08-024, B-08-029, and B-08-053), nor was a resistance marker detected by the PN panel in three specimens containing an *Enterobacter* sp. (B-05-012) or *Klebsiella* spp. (B-01-040 and B08-027) that were reported as ESBL positive based on phenotypic test results. These data demonstrate a combined 20% (2/10) concordance between the identification of genetic resistance markers by the PN panel and the phenotypic characterization of Gram-negative isolates recovered from these specimens.

**TABLE 5:** Comparison of BioFire PN panel and phenotypic results for detection of carbapenemase and ESBL-producing organisms^*a*^

Specimen	PN panel bacterial target detected	PN panel resistance marker detected	Culture result	Phenotypic susceptibility (method)
B-08-008	A. baumannii, 10^6^	*bla*_KPC_	A. baumannii, “few”	Carbapenem resistant (MIC)
	K. pneumoniae, 10^4^		ND	NA
B-03-008	E. cloacae, 10^6^	*bla*_KPC_	E. cloacae, ≥10^5^	Carbapenem resistant (MIC), ESBL positive (Etest ESBL)
	P. aeruginosa, ≥10^7^		P. aeruginosa, ≥10^5^	Phenotypic tests not performed
B-01-015	E. cloacae, 10^4^	*bla*_NDM_	E. cloacae, 10^3^	Carbapenem susceptible (MIC), ESBL negative (BMD)
B-08-056	P. aeruginosa, 10^6^	*bla*_CTX-M_	P. aeruginosa, “moderate”	Carbapenem resistant (MIC)
B-01-040	K. pneumoniae, 10^6^	ND	K. pneumoniae, 10^4^	Carbapenem susceptible (MIC), ESBL positive (BMD)
B-05-012	E. cloacae, 10^5^	ND	E. cloacae, ≥10^5^	Carbapenem not tested, ESBL positive (KB)
B-08-024	P. aeruginosa, ≥10^7^	ND	P. aeruginosa, “few”	Carbapenem resistant (MIC)
B-08-027	K. oxytoca, 10^4^	ND	K. oxytoca, “rare”	Carbapenem susceptible (MIC), ESBL positive (MIC)
B-08-029	P. aeruginosa, ≥10^7^	ND	P. aeruginosa, “few”	Carbapenem resistant (MIC)
B-08-053	P. aeruginosa, ≥10^7^	ND	P. aeruginosa, “moderate”	Carbapenem resistant (MIC)

a ND, not detected; KB, Kirby-Bauer disk diffusion test; BMD, broth microdilution test; Etest ESBL, Etest ESBL test strip.

### Qualitative agreement of viral targets with standard-of-care methods.

Among 259 BAL specimens tested, the PN panel detected a total of 53 viral targets in 46 unique specimens (Table 6). The most commonly detected target was human rhinovirus/enterovirus (*n* = 21), followed by coronavirus (*n* = 12) and influenza A virus (*n* = 6). A SOC test for viral agents was ordered for only 90/259 (34.7%) specimens. Among these, the PN panel demonstrated 96.7% (87/90) agreement with the SOC result, with no false-positive detections. Three discordant results included one specimen reported as positive for parainfluenza virus 3 by the BioFire RP panel, one specimen positive for influenza A virus by the Xpert Flu/RSV XC assay (Cepheid, Sunnyvale, CA), and one specimen positive for adenovirus by the GenMark XT-8 Respiratory Viral panel that were reported as “not detected” by the PN panel. Of note, SOC data were abstracted from the medical record to include all test results reported within 24 h of the specimen’s being tested by the PN panel. Therefore, it is possible that the discordant results were obtained from SOC testing of a different specimen or specimen type than that tested by the PN panel (e.g., a nasopharyngeal swab collected the same day as the BAL specimen).

**TABLE 6:** Qualitative comparison of viral targets detected by BioFire PN panel and standard-of-care testing in 259 BAL specimens^*a*^

Target(s) detected by BioFire PN panel	No. of BAL specimens	No. (%) of BAL specimens with SOC test order for viral target(s) detected	Agreement (%) between SOC and PN panel for viral target(s) detected	No. (%) of BAL specimens with bacterial target codetection by PN panel
hRV/EV	17	6 (35)	6/6 (100)	7 (41)
CoV	9	2 (22)	2/2 (100)	2 (22)
FluA	5	0 (0)	NA	2 (40)
FluB	2	1 (50)	1/1 (100)	1 (50)
RSV	2	0 (0)	NA	0 (0)
PIV	3	1 (33)	1/1 (100)	1 (33)
hMPV	1	0 (0)	NA	1 (100)
AdV	1	0 (0)	NA	0 (0)
CoV+hMPV	1	1 (100)	1/1 (100)	1 (100)
hRV/EV+PIV	3	0 (0)	NA	2 (66)
hRV/EV+CoV	1	0 (0)	NA	1 (100)
hMPV+FluA+CoV	1	0 (0)	NA	0 (0)
None detected	213	79 (37)	76/79^*b*^ (96)	80 (38)
Total	259	90 (34.7)	87/90 (96.7)	98 (37.8)

a BAL, bronchoalveolar lavage; SOC, standard-of-care molecular test; hRV/EV, human rhinovirus/enterovirus; CoV, coronavirus; FluA, influenza A virus; FluB, influenza B virus; RSV, respiratory syncytial virus; PIV, parainfluenza virus; hMPV, human metapneumovirus; AdV, adenovirus.

b Three BAL specimens were reported as positive for an on-panel target by the standard-of-care test that was not detected by the BioFire PN panel. This included one specimen reported as positive for PIV-3 (BioFire FilmArray Respiratory panel), one specimen positive for FluA (Cepheid Xpert Flu/RSV assay), and one specimen positive for AdV (GenMark XT-8 system).

Importantly, only 23.9% (11/46) of all specimens with a positive PN panel result had a standard-of-care order for viral pathogen testing. The lack of SOC comparator test orders for the majority of specimens enrolled limits the ability to assess sensitivity and specificity of the PN panel for viral targets. However, it highlights an apparent underappreciation of the prevalence of viral pathogens in this patient population, which may result in delayed or missed diagnosis, missed opportunity to de-escalate antibiotics, and missed opportunity for the implementation of appropriate infection prevention strategies within the hospital ICU setting. Specifically, only 1/7 specimens testing positive for influenza virus, 2/9 testing positive for human coronavirus, 1/3 testing positive for parainfluenza virus, and 6/17 testing positive for human rhinovirus/enterovirus by the PN panel had a physician-ordered SOC test capable of detecting these viruses.

### Potential impact of the BioFire PN panel on antibiotic utilization.

Complete medical chart data were available for 253/269 (94.1%) patients whose specimens were included in this study. Potential antibiotic adjustments were based on comparison between the PN panel and routine culture results. Actual antibiotic prescription and modification based on the SOC results were determined by medical chart review. Potential antibiotic adjustments based on the PN panel results were considered appropriate only if PN panel and SOC results were in positive or negative agreement. In these cases, it was assumed that the same appropriate adjustment could have been made at the time of PN panel result. Potential antibiotic adjustments based on PN panel results were considered “inappropriate” if results between PN panel and SOC were discordant (see Table S1 and Materials and Methods for specific criteria).

There was a potential for antimicrobial modification based on PN panel results for 179/253 (70.8%) of evaluable patients (Table 7). For many patients, there was an opportunity for multiple antimicrobial modifications, including simultaneous escalation and de-escalation, due to the empirical utilization of multiple antibiotics in a single patient (average of 1.48 potential modifications per patient).

**TABLE 7:** Potential impact of the BioFire PN panel result on antibiotic utilization

Potential modification	No. of antimicrobials	No. (%) of patients	No. of hrs
Appropriate de-escalation/discontinuation	206	122 (48.2)	18,284.07
Appropriate escalation/initiation	11	11 (4.3)	184.66
Inappropriate de-escalation/discontinuation	4	4 (1.6)	
Inappropriate escalation/continuation	42	42 (16.6)	
No change		74 (29.2)	
Unable to assess^*a*^		16	

a No stop date was listed for antimicrobials, concomitant infection was present, or antimicrobials were used for longer durations than would be used for a lower respiratory tract infection (>30 days).

**FIG 4:**
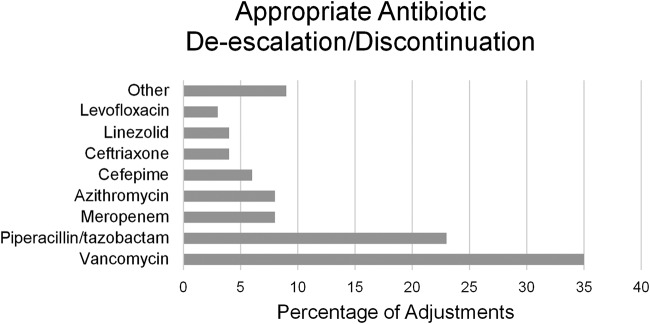
Percentage of total antibiotic de-escalations and discontinuations among 122 patients with negative agreement between SOC and PN panel results. Vancomycin and piperacillin-tazobactam accounted for 58% of total antimicrobial de-escalations and discontinuations based on negative results for MRSA and *Enterobacteriaceae*, respectively.

The most common potential intervention was an appropriate antibiotic de-escalation or discontinuation, which encompassed 206 total antimicrobials (78.3% of all antimicrobials considered) in 122 individual patients (48.2% of all patients considered). These de-escalations and discontinuations resulted in a potential to reduce the duration of unnecessary antibiotic therapy by over 18,000 cumulative hours in these 122 patients. On a per-patient basis, this equated to an average potential of 6.2 fewer total antibiotic days per patient or 3.7 days per antibiotic. PN panel results most commonly allowed de-escalation or discontinuation of vancomycin (38% of de-escalations/discontinuations) and piperacillin-tazobactam (23% of de-escalations/discontinuations) due to negative results for MRSA and Gram-negative bacilli, respectively (Fig. 4).

Appropriate escalation or initiation of antibiotics would have been possible in 11/253 (4.3%) of patients based on positive agreement between PN panel and SOC results, enabling initiation of active antibiotics a combined total of 184.7 h earlier than routine culture results for these patients. Specifically, for 4 of the 11 patients, PN panel results would have resulted in reduced time to appropriate Gram-negative coverage for patients with pneumonia secondary to Gram-negative organisms inadequately covered by the empirical antibiotic regimen. In 1/11 patients, PN panel results would have prompted MRSA coverage to be initiated sooner. Finally, in 6/11 patients, the PN panel detected influenza A or B virus when no clinician order for an influenza virus test had been placed. This would have enabled rapid initiation of antiviral therapy as well as droplet isolation, neither of which was implemented for these patients.

The PN panel result might have alternatively prompted inappropriate antimicrobial de-escalation or discontinuation in four patients. For three of these patients, the PN panel failed to detect an organism isolated by routine culture methods. Of note, in all cases, the quantity of the organism reported in routine culture was below the threshold for clinical significance recommended by current guidelines (10^3^ CFU/ml or “few”) (Table 1) and was likely below the designed lower limit of detection of the PN panel. Despite this, it is possible that acting upon the negative PN panel result would have resulted in inappropriate de-escalation of antimicrobials and potential undertreatment if the organisms were of clinical relevance. For the remaining patient, routine culture yielded 1.5 × 10^4^ CFU/ml methicillin-resistant S. aureus (MRSA). The PN panel identified S. aureus (10^5^ copies/ml) but failed to detect *mecA/mecC and* MREJ.

There were 38 culture-negative specimens with at least one bacterial target detected by the PN panel (31 with single targets and 7 with multiple targets). In the majority of these cases (23/38; 60.5%), SOC reported “normal oral flora.” Due to laboratory-specific culture reporting protocols, identification of individual bacteria present in these specimens was not completed. Furthermore, 11/38 (28.9%) targets were quantified at 10^4^ copies/ml by the PN panel, indicating a low organism burden. In these specimens, utilization of the PN panel result might have resulted in inappropriate initiation or escalation/continuation of antimicrobials that were not prescribed based on culture results.

Finally, for 80/256 (31.6%) of patients, PN panel and SOC were in either positive or negative agreement and the patient was receiving antibiotic therapy at the time of sample collection that was determined to be optimal for the identified organisms. Therefore, no antibiotic modification would have been made based on the PN panel result.

## DISCUSSION

This study was specifically designed to provide a pragmatic assessment of the PN panel, including a comparison to routine laboratory test results and the potential impact of results on antibiotic therapy. As such, PN panel results were compared to the standard-of-care method(s) employed at each participating laboratory rather than a standardized culture protocol. This included a comparison to any culture-based or molecular tests ordered for clinical care within 24 h preceding to 48 h following collection of the specimen tested with the PN panel. No additional tests were conducted on specimens to resolve discrepant results or provide a comparator if a clinical test order was not received (e.g., the PN panel detected influenza A virus but no SOC test for respiratory viruses was ordered). A comprehensive analytic evaluation of the PN panel study is provided by Murphy et al. (14).

Compared to SOC bacterial culture methods, use of the PN panel resulted in a 63.3% increase in specimens reported as positive and a 94.8% increase in the total number of bacterial targets detected. These specimens were reported as “negative/no growth” (39.5%) or “negative/normal oral flora” (60.5%) based on routine culture and reporting protocols. We did not perform additional tests to confirm the accuracy of PN panel results in culture-negative specimens; however, >99% of culture-negative discordant results were found to be positive using an alternative molecular test or were below the culture threshold for reporting during the clinical trial for regulatory clearance, suggesting that these are not false-positive detections (14). In addition, our results are comparable to findings published by Lee et al., who reported a 70.3% increase in total bacterial targets detected by the PN panel among 59 BAL and endotracheal aspirate specimens (19), and Ozongwu et al., who reported a 63.4% increase in positive specimens and a 129.4% increase in total bacterial targets detected using different multiplex molecular assays (20). The increase in positive results reported by molecular tests is not unexpected due to the detection of both viable and nonviable organisms, as well as detection of low-abundance targets and those not recovered in culture due to fastidious growth characteristics. However, the interpretation and potential significance of these results require special attention to determine the impact of reporting on patient management.

In our study, approximately one-quarter (20/73; 27.4%) of the culture-negative detections were quantified as 10^4^ copies/ml by the PN panel. Semiquantitative values reported (in copies per milliliter) by the PN panel are on average approximately 1 log_10_ higher than values (CFU per milliliter) reported from culture. This quantitative discordance was noted in our study (discussed below) as well as in other preliminary evaluations of the PN panel (19, 21, 22). Therefore, targets quantified as 10^4^ copies/ml by the PN panel may frequently be quantified as 10^3^ CFU/ml by routine culture. This would approach the culture-based limit of detection of 10^3^ CFU/ml if one is relying on the use of a 1-μl loop for specimen inoculation. Furthermore, potential pathogens present at <10^4^ CFU/ml in culture may not be routinely reported in accordance with current guidelines (3, 12). Studies comparing the clinical outcomes of patients stratified by culture-based quantification of bacterial targets have demonstrated no increase in mortality for patients with bacteria quantified below 10^4^ CFU/ml in BAL specimens (23). The clinical significance of additional culture-negative low-concentration (10^4^ copies/ml) detections by the PN panel requires further investigation, especially given that these detections have historically gone largely unreported based on current culture and reporting guidelines. In the absence of these studies, such results should be interpreted with caution and in the context of other laboratory results (e.g., additional pathogens present at high concentrations in the same specimen) and clinical symptoms.

Another potential factor resulting in culture-negative, PN panel-positive results is the use of empirical antibiotics prior to specimen collection. Exposure to antibiotic therapy for as little as 1 h prior to collection can dramatically reduce culture-based recovery of potential pathogens from clinical specimens (24–26). Up to 80% of PCR-positive, culture-negative results may be attributable to recent exposure to empirical antibiotics, underscoring both the frequency of empirical antibiotic use in these patients and the negative consequence for culture-based diagnosis (27, 28). In our study, nearly 50% (36/73) of culture-negative detections by the PN panel were in specimens obtained from patients who received antibiotic therapy within 72 h preceding specimen collection. The PN panel quantitation of these targets ranged from 10^4^ to >10^7^ copies/ml, including 28/36 (77.8%) reported at ≥10^5^ copies/ml. These notably included S. aureus (*n* = 7), H. influenzae (*n* = 7), S. pneumoniae (*n* = 3), and P. aeruginosa (*n* = 2), among others. Unlike culture-negative targets quantified as 10^4^ copies/ml, these may be more likely to represent clinically significant infections and would have been more likely to be recovered and reported by routine culture had the patient not received preemptive antibiotics. The potential benefit of these PN panel detections is 2-fold. First, detection of a high-concentration (clinically significant) pathogen could potentially prevent the early termination of effective antibiotics. Current guidelines recommend a 7-day antibiotic course for treatment of HAP and VAP (3). A negative culture result at 72 h may result in premature discontinuation and risk of relapse. Second, identification of a specific pathogen(s) could enable modification (escalation or de-escalation) of empirical antibiotic therapy even in the face of a negative culture. For example, a PN panel result of H. influenzae or S. pneumoniae could enable de-escalation of empirical broad-spectrum agents, such as vancomycin and cefepime or meropenem, to a more narrow-spectrum beta-lactam regimen, such as ceftriaxone or amoxicillin. Appropriate narrowing or discontinuation of antibiotic therapy based on quantitative culture results has been associated with a decrease in subsequent infection with multidrug-resistant organisms (MDROs) (23). Early identification of bacterial pathogens by the PN panel, even in negative cultures, may have a similar impact. Confirmation of these hypotheses would require a longitudinal comparison of patients whose antibiotics were withheld, modified, or continued broadly based on a positive PN panel but negative culture result. Unfortunately, the design of our study did not allow the collection of these data, and this remains an area of significant interest requiring research.

Quantitative reporting of bacterial counts in BAL specimens has been recommended to aid in the interpretation of results (3). The importance of quantitative molecular reporting for bacterial pathogens in respiratory specimens was demonstrated by a recent study evaluating a multiplex molecular test that reports only qualitative results. Compared to quantitative 16S next-generation sequencing (NGS) analysis, targets identified by the qualitative test spanned a wide range from 0.5% to 95.7% of all NGS reads obtained from the specimen (20). Targets with a low percentage of reads frequently were not recovered in culture, leading to better agreement between the quantitative molecular approach (NGS) and culture. Detection of low-abundance organisms or an inability to differentiate predominant organisms in polymicrobial cultures may limit the ability to appropriately modify antibiotic therapy or result in treatment of clinically insignificant colonizing bacteria (3, 13). Our quantitative comparison between PN panel and culture results was somewhat limited by differences in the range of values reported by each method. The PN panel has a broad dynamic range and assigns a bin value of 10^4^, 10^5^, 10^6^, or ≥10^7^ copies/ml, whereas routine SOC culture reports values spanning a narrower range, including 10^3^, 10^4^, or ≥10^5^ CFU/ml. Therefore, a direct log_10_ bin comparison was possible only for values reported at 10^4^ copies or CFU/ml. Values of 10^3^ CFU/ml in culture were considered concordant if reported as “not detected” by the PN panel, and values of ≥10^5^ CFU/ml in culture were considered concordant if reported as 10^5^, 10^6^, or ≥10^7^ copies/ml by the PN panel. Based on these criteria, there was only 43.6% “log_10_ bin” quantitative concordance between the PN panel and SOC for culture-positive specimens. A 100% (27/27) concordance was observed for targets reported as ≥10^5^ CFU/ml in culture (10^5^, 10^6^, or ≥10^7^ copies/ml by the PN panel), while log_10_ bin concordance was just 11% to 19% for targets reported at 10^3^ or 10^4^ CFU/ml, respectively. The relatively poor absolute quantitative correlation between the PN panel (copies per milliliter) and culture (CFU per milliliter) results was noted in the clinical trial data set and is acknowledged as a limitation in the PN panel product labeling (14). However, if a categorical-agreement model for treatment based on suggested thresholds of 10^4^ CFU/ml for BAL is considered, then the PN panel and culture demonstrate 82.7% (62/75) categorical agreement with no very major errors; i.e., PN panel quantification was never <10^4^ copies/ml when culture results were ≥10^4^ CFU/ml.

In addition to absolute quantitation of individual targets, the relative abundance of each organism in a polymicrobial specimen may be of value when therapy is being targeted to the most likely pathogen (13). To that end, we examined the agreement between the PN panel and culture for reporting the same organism as predominant (i.e., most abundant) in a clinical specimen. Among culture-positive specimens, we observed over 93% agreement between the PN panel and culture for this comparison. Taken together, these data demonstrate excellent categorical and relative abundance agreement between the PN panel and culture methods despite low absolute quantitative correlation (i.e., same log_10_ value). When the clinical utility of the PN panel result is being considered, relative abundance and predominance of a given organism are more likely to drive patient management based on current guidelines than the exact quantitative value. However, it will be important to educate clinicians on the expected differences in absolute value observed between culture results in CFU per milliliter and the PN panel results in copies per milliliter to avoid potential confusion when these values are compared and applied to current culture-based guidelines.

Molecular detection of genetic markers associated with antibiotic resistance, including *mecA*, carbapenemases, and ESBLs, has been associated with positive outcomes, including reduced time to optimal antibiotic therapy, shorter length of ICU stay, and reduced mortality (29, 30). Furthermore, many health care systems employ specific contact isolation policies for patients harboring resistant bacteria containing these markers. Among 21 specimens that were culture-positive for S. aureus, the PN panel demonstrated a modest 81.1% sensitivity and 85.7% specificity for identification of MRSA based on the detection of *mecA/mecC* and MREJ. False-negative results may represent MRSA isolates that contain divergent sequences within the MREJ region targeted by the PN panel. Alternatively, these cultures may contain a mixture of MRSA and MSSA, with MRSA falling below the PN panel limit of detection (LoD). Specimens containing S. aureus with the MREJ sequence but lacking *mecA* (i.e., “*mecA* dropout”), in addition to methicillin-resistant coagulase-negative *Staphylococcus* spp., could result in an apparent false-positive result. Each of these scenarios was encountered during the clinical trial, which reported a higher postresolution sensitivity of 88.9% and specificity of 91.4% for detection of MRSA in BAL specimens (14). Importantly, our cohort included just 21 specimens that were culture positive for S. aureus. Evaluation of a larger number of MRSA-positive specimens and characterized isolates is needed to provide a more complete assessment of the PN panel for the identification of MRSA. Nonetheless, a positive *mecA*/MREJ detection by the PN panel was reported for 8/21 (38.1%) of culture-negative specimens. Identification of MRSA in these additional patients has the potential to ensure appropriate antibiotic therapy in patients whose therapy might have otherwise been de-escalated based on negative cultures. Furthermore, while negative culture results likely limit the risk of transmission, detection of MRSA also enables implementation of infection prevention practices in accordance with existing hospital policies.

A low incidence of phenotypic carbapenem resistance was observed in this study. Only 6/259 (2.3%) specimens contained isolates that exhibited MICs indicating carbapenem resistance, including P. aeruginosa (*n* = 4), Acinetobacter baumannii (*n* = 1), and E. cloacae (*n* = 1). The PN panel detected specific carbapenemase genes in the 2 specimens containing carbapenem-resistant E. cloacae or A. baumannii; however, no carbapenemase genes were detected by the PN panel in the 4 specimens containing carbapenem-resistant P. aeruginosa. While we cannot rule out a false-negative PN panel result for carbapenemase detection, carbapenem resistance in P. aeruginosa is most often mediated by mechanisms other than carbapenemases (e.g., efflux pumps or expression of altered outer membrane porins) (31). Therefore, molecular detection of carbapenemase-encoding genes in non-*Enterobacteriaceae* has a limited negative predictive value. Similarly, detection of *bla*_CTX-M_ demonstrated a 100% positive predictive value but a reduced negative predictive value for predicting the presence of ESBL-producing bacteria in BAL specimens due to the diversity of enzymes capable of conferring this phenotype. These are important limitations that extend to all molecular diagnostic tests that target specific genetic markers to predict phenotypic susceptibility. Therefore, failure to detect a specific gene should not be interpreted as phenotypic susceptibility. Conversely, detection of a specific genetic marker carries a high probability of phenotypic resistance to the corresponding antibiotic class. These detections can aid in both rapid antibiotic escalation when indicated as well as implementation of appropriate infection prevention practices for these patients.

The role of viral pathogens in hospital-acquired pneumonia (HAP) and ventilator-associated pneumonia (VAP) has only recently been appreciated, in part due to the increased availability of multiplex molecular panels to detect these agents. A recent study identified a viral pathogen in 22.4% of patients being evaluated for HAP with the risk of a viral infection increasing with duration of ICU stay (32). Interestingly, no seasonal trend in positivity was noted, suggesting that viruses should be routinely considered in this patient population. In our study, the PN panel detected a viral pathogen in approximately 18% of specimens tested. Human rhinovirus/enterovirus was the most frequently detected viral target, but all 9 PN panel viral targets were identified in at least one clinical specimen. Of interest, only one-third of specimens had a clinical test order for viral pathogens, including only one-quarter of specimens with a virus detected by the PN panel. The lack of a uniform SOC comparator test order and result for the majority of enrolled specimens precludes a thorough comparative assessment of the PN panel performance for detection of viral targets; however, the clinical trial demonstrated >93% positive agreement and >99% negative agreement between the PN panel results and reference PCR and sequence analysis for each target (14). Importantly, our data demonstrate the infrequency of clinician orders for tests to detect common respiratory viruses in this patient population, which could contribute to underdiagnosis or delayed diagnosis of potentially treatable infections. Specifically, only 1/7 BAL specimens that were reported as positive for influenza A or B virus by the PN panel had a standard-of-care order for a test capable of detecting these pathogens. Early recognition of influenza virus infections might have resulted in administration of antiviral therapy that could have shortened the duration and severity of symptoms in these patients (33, 34). While no specific therapy is available for the majority of viral pathogens, 60.9% (28/46) of specimens with a viral detection did not have a bacterial target codetection, supporting the potential contribution of these viruses to respiratory symptoms observed in these patients. In these patients, definitive identification of a viral agent in the absence of a bacterial pathogen could enable an opportunity for antibiotic stewardship. Furthermore, patients with underlying comorbidities are at risk of severe infection by human metapneumovirus, parainfluenza viruses, and others (35, 36). Recognition of these infections in ICU patients enables appropriate infection prevention practices, including droplet isolation to prevent subsequent hospital-acquired infections in a susceptible population. Combined, these results support the added value of viral targets as part of multiplex panels designed to aid in diagnosis of lower respiratory tract infections, including HAP and VAP.

A major goal of our study was to examine the potential impact of the PN panel results on antibiotic utilization. For specimens with positive or negative agreement between the PN panel and routine culture, it was assumed that the antibiotic modifications made based on routine culture results would also be made based on the PN panel result. Based on review of patient charts, we found that antibiotic adjustments could have been made for over 70% of patients who submitted respiratory specimens for this study. Most commonly, this involved discontinuation or de-escalation of empirical therapy and encompassed approximately 80% of all antimicrobial modifications and nearly 50% of patients included in the analysis. Combined, this equated to a total of >18,000 h of antibiotic sparing, or an average of 3.8 days/antibiotic. This 3.8-day differential essentially equates to the time difference between availability of the PN panel result and the final culture result. Importantly, while it was less common, we did find 4.3% of patients with results indicating that they were receiving ineffective antibiotic therapy for pathogens or resistance markers detected by the PN panel and ultimately reported by routine culture. Notably, 6/11 of these were detection of influenza A or B virus by the PN panel in patients without a clinician-ordered test capable of detecting these viruses. Early recognition of antibiotic mismatches can be potentially lifesaving, as demonstrated by an increased mortality rate observed for patients receiving ineffective or delayed therapy (37, 38). As discussed above, it is difficult to determine the clinical significance of PN panel detections in culture-negative specimens. For this analysis, we chose to be conservative and consider these “false-positive” PN panel results potentially leading to inappropriate or unnecessary antibiotic initiation/escalation. Using these criteria, approximately 17% of patients reviewed would have received antibiotics that were not prescribed based on routine culture results. Importantly, the PN panel is an adjunct to early decision-making and does not replace clinical assessment and review of other laboratory values. It is reasonable to assume that a single target present at 10^4^ copies/ml in a patient with minor clinical symptoms or alternative etiology might not result in antibiotic treatment based solely on the PN panel result. However, using conservative criteria assuming that all detections lead to antibiotic prescriptions, the overall data still demonstrate a 3:1 ratio of appropriate to inappropriate antibiotic modifications based on PN panel results. Of note, antibiotic therapy for 29% of patients would not have been modified based on the PN panel result; however, early confirmation that these patients were receiving optimal therapy might also have been beneficial in managing care.

This study did have limitations. First, it was assumed that all specimens were obtained from patients with clinical symptoms concerning for pneumonia. It is possible that some of the specimens could have been collected as part of surveillance protocols from asymptomatic patients who had recently undergone transplant procedures. Second, PN panel results were compared to the routine SOC methods and results reported by seven different clinical laboratories, each of which used slightly different specimen processing, plating, and reporting protocols. Furthermore, we relied solely on results of clinician-ordered tests, which were absent for approximately 80% of specimens in the context of viral etiologies. A full clinical and analytical evaluation is reported elsewhere (14) but does not address how PN panel results compare to SOC methods currently used in clinical laboratories. This study was specifically designed to be a pragmatic comparison of PN panel results to SOC test results to examine how implementation of the PN panel might impact qualitative positivity and quantitative result reporting. Our data raise awareness of specific aspects of the PN panel results, including qualitative and quantitative concordance as well as identification of additional bacterial and viral pathogens that can be expected following implementation of the PN panel, so that laboratory directors and clinicians can begin to discuss utilization and result reporting based on this novel test approach. One of the key findings was the potential for a large impact on antibiotic adjustments, primarily de-escalation and discontinuation, based on the PN panel result. These results were similarly limited by the retrospective design of the study; however, we took a conservative approach, considering adjustments appropriate only when positive or negative agreement was observed between PN panel and SOC results. The actual impact of these potential adjustments on quality indicators such as length of stay, 30-day mortality, and readmission rate could not be assessed based on retrospective analysis and will require prospective randomized controlled trials. Still, the potential for early recognition and de-escalation or discontinuation of antibiotics in specimens that test negative by PN panel and subsequent routine culture is attractive for meeting antibiotic stewardship goals.

Notably, we considered only adult inpatients and only BAL or mini-BAL specimens in this study. Therefore, we have not evaluated the potential impact of the PN panel results in pediatric or ambulatory outpatient populations or with other validated specimen types, such as sputum or sputum-like (e.g., endotracheal aspirate) specimens. Given the greater acuteness of illness in hospitalized patients, we chose to focus on the benefit of the PN panel in this population. Similarly, we chose to focus on BAL specimens, because they are frequently of better quality and results have a higher predictive value for lower respiratory tract infection than those obtained with sputum specimens, which may more frequently contain higher bacterial burdens and diversity of insignificant upper airway or oral flora. Preliminary data (42, 43) did demonstrate a higher percentage of positive specimens among sputum specimens than BAL specimens, though clinical data and the impact of these detections on antibiotic stewardship or potential patient outcome were not assessed. Therefore, there remains a need for similar studies focusing on these alternative populations and specimen types.

In conclusion, we present the first “real-life” assessment of the potential impact of the PN panel results on reporting compared to routine SOC culture and clinician-ordered viral PCR tests, as well as the potential for antibiotic stewardship based on these results. Laboratories considering implementation of the PN panel will have to give careful consideration to reporting and interpretation of results because of the increased sensitivity and differences in absolute quantification between PN panel and SOC methods. Clinician education will also be an important component of successful implementation of the PN panel to ensure that results are interpreted in the context of other laboratory results. Laboratories may choose to report discrete semiquantitative bin values with clinician understanding that these will be higher than current culture-based quantitative results. Alternatively, laboratories may choose to report relative abundance of bacterial targets based on PN panel results, assigning each log bin a numeric value from 1 to 4 so that the clinician can quickly assess the predominant pathogen(s) in the specimen. Likewise, education focused on the interpretation of results of genotypic resistance, including the positive and negative predictive value of markers, will be important to maximize antibiotic stewardship efforts and ensure appropriate adjustments. Specifically, negative results do not preclude phenotypic resistance due to alternative mechanisms. As with other rapid diagnostic tests, maximizing the impact of PN panel results involves the utilization of additional resources, including electronic decision support to ensure appropriate utilization, inclusion of interpretive comments in the lab report to aid interpretation, and inclusion of an active interventional antimicrobial stewardship team (39–41). The PN panel will not be used as a replacement for routine culture. However, as an adjunctive test for patients with symptoms of lower respiratory tract infection, the PN panel has the potential to provide rapid identification of bacterial and viral pathogens that can be used to aid in definitive etiologic diagnosis and positively impact efforts to meet infection prevention and antibiotic stewardship objectives.

## Supplementary Material

Supplemental file 1
