# Supplementary material for: Practical Comparison of the BioFire FilmArray Pneumonia Panel to Routine Diagnostic Methods and Potential Impact on Antimicrobial Stewardship in Adult Hospitalized Patients with Lower Respiratory Tract Infections
Source: J Clin Microbiol. 2020 Jun 24;58(7):e00135-20. doi: 10.1128/JCM.00135-20 (PMC7315039; doi:10.1128/JCM.00135-20)
Supplement: Supplemental file 1 [file JCM.00135-20-s0001.pdf]

Supplementary Table S1. Criteria used to define potential antibiotic adjustments

Potential antimicrobial therapy modification based on BioFire PN Panel Result	Description
Appropriate antimicrobial escalation	On-panel organism(s) or resistance mechanism(s) were identified by **both** SOC and PN Panel, i.e. positive agreement<br><br>• The PN Panel would enable more rapid **initiation** of appropriate antimicrobials<br>➢ e.g. *P. aeruginosa* identified by the PN Panel and SOC in a patient receiving ceftriaxone and azithromycin. Patient could be escalated to anti-pseudomonal agent based on the PN Panel result.
Appropriate antimicrobial de-escalation/ discontinuation	No on-panel organism(s) or resistance mechanism(s) identified be either SOC or PN Panel. i.e. negative agreement<br><br>• The PN Panel would enable more rapid **discontinuation** of one or more antimicrobial agents<br>➢ e.g. *K. pneuomoniae* detected by the PN Panel and SOC in a patient receiving cefepime and vancomycin. Vancomycin could be discontinued sooner based on the PN Panel result negative for *S. aureus*.<br><br>• The PN Panel would enable more rapid **de-escalation** of one or more antimicrobial agents<br>➢ e.g. Methicillin-susceptible *S. aureus* detected by the PN Panel and SOC in a patient receiving vancomycin. Vancomycin could be de-escalated sooner to cefazolin based on FA result.
Inappropriate antimicrobial escalation/continuation	On-panel organism(s) or resistance mechanism(s) were identified by the PN Panel but not SOC, i.e. PN Panel false-positive<br><br>• The PN Panel may result in inappropriate **initiation or escalation** of one or more antimicrobial agents<br>➢ e.g. *S. aureus* and *mecA/MREJ* detected by the PN Panel but not SOC in a patient receiving ceftriaxone. Vancomycin may be initiated/continued despite eventual negative culture result.
Inappropriate antimicrobial de-escalation/ discontinuation	On-panel organism(s) were identified by SOC but not the PN Panel, i.e. PN Panel false-negative<br><br>• The PN Panel may result in inappropriate **discontinuation or de-escalation** of one or more antimicrobial agents<br>➢ e.g. S. aureus identified in SOC but not by the PN Panel in a patient receiving vancomycin. Vancomycin may be discontinued based on negative the PN Panel result until SOC results become available.
No therapy change	Concordant SOC and the PN Panel results, i.e. positive or negative agreement<br>• Empirical antibiotics appropriate for identified organism(s), **no escalation or de-escalation** possible based on the PN Panel result
